# Plants *versus* Fungi and Oomycetes: Pathogenesis, Defense and Counter-Defense in the Proteomics Era

**DOI:** 10.3390/ijms13067237

**Published:** 2012-06-13

**Authors:** Abdelbasset El Hadrami, Ahmed F. El-Bebany, Zhen Yao, Lorne R. Adam, Ismailx El Hadrami, Fouad Daayf

**Affiliations:** 1 Department of Plant Science, University of Manitoba, 222, Agriculture Building, Winnipeg, Manitoba, R3T 2N2, Canada; E-Mails: abdele@omexcanada.com (A.E.H.); aelbebany@yahoo.com (A.F.E.-B.); yao@cc.umanitoba.ca (Z.Y.); lorne_adam@umanitoba.ca (L.R.A.); 2 OMEX Agriculture Inc., P.O. Box 301, 290 Agri Park Road, Oak Bluff, Manitoba, R0G 1N0, Canada; 3 Department of Plant Pathology, Faculty of Agriculture, Alexandria University, El-Shatby, Alexandria, 21545, Egypt; 4 Laboratoire de Biotechnologies, Protection et Valorisation des Ressources Végétales (Biotec-VRV), Faculté des Sciences Semlalia, B.P. 2390, Marrakech, 40 000, Morocco; E-Mail: hadrami@hotmail.com

**Keywords:** biotrophs, counter-defenses, defenses, effectors, hemibiotrophs, necrotrophs, pathogenicity factors, plant-pathogen interactions, pathosystems, proteome, proteomics approach

## Abstract

Plant-fungi and plant-oomycete interactions have been studied at the proteomic level for many decades. However, it is only in the last few years, with the development of new approaches, combined with bioinformatics data mining tools, gel staining, and analytical instruments, such as 2D-PAGE/nanoflow-LC-MS/MS, that proteomic approaches thrived. They allow screening and analysis, at the sub-cellular level, of peptides and proteins resulting from plants, pathogens, and their interactions. They also highlight post-translational modifications to proteins, *e.g.*, glycosylation, phosphorylation or cleavage. However, many challenges are encountered during *in planta* studies aimed at stressing details of host defenses and fungal and oomycete pathogenicity determinants during interactions. Dissecting the mechanisms of such host-pathogen systems, including pathogen counter-defenses, will ensure a step ahead towards understanding current outcomes of interactions from a co-evolutionary point of view, and eventually move a step forward in building more durable strategies for management of diseases caused by fungi and oomycetes. Unraveling intricacies of more complex proteomic interactions that involve additional microbes, *i.e.*, PGPRs and symbiotic fungi, which strengthen plant defenses will generate valuable information on how pathosystems actually function in nature, and thereby provide clues to solving disease problems that engender major losses in crops every year.

## 1. Introduction

Plant-pathogen interactions are among the most complex and exciting associations to study. They involve living organisms and counterparts able to generate and evolve various infection and pathogenesis strategies. Through their co-evolution, both plants and their pathogens have shaped their strategies to survive and eventually win the fight against their respective invader or host. This occurs through a constant battle involving an array of pathways and signaling networks that shape pathogenesis, plant defenses and further pathogen counter-defenses [1,2]. Proteomic studies during plant-pathogen interactions are crucial because most of the pathways used either by plants or pathogens rely on protein synthesis and activity. These have been pursued for many decades at a slow pace and have led to the characterization of many families of the so-called Pathogenesis Related-proteins (PRs) involved in plant defenses [3], or others involved in pathogenesis [1]. In recent years, new bioinformatics mining tools have emerged, thereby accelerating the discovery and annotation of such proteins [2]. The 2-dimensional polyacrylamide gel electrophoresis (2D-PAGE) technology was combined with new analytical instruments such as nanoflow liquid chromatography coupled to tandem mass spectrometry (*i.e.*, LC-MS/MS) to establish proteome maps and explain specific aspects of host-pathogen interactions [4,5]. Other approaches that do not require the use of PAGE have been developed [6] and searchable databases have been increasingly updated with newly discovered proteins based on Molecular Weight (MW), Isoelectric Focusing point (IEF) and characteristic MS/MS spectra of *m/z* (mass over charge) [5]. Cellular and sub-cellular screenings and other *in silico* annotations or *in vitro* functional analyses were also conducted for peptides and proteins originating from the plant, the pathogen or their interactions [2]. Through proteomics, it is also possible to reveal post-translational modifications in proteins after their synthesis [7]. These include compartment signal peptide cleavages, glycosylation and deglycosylation, phosphorylation and dephosphorylation as well as other unfolding modifications. Many mechanisms of the interplay between signaling networks, on the other hand, remain to be elucidated to determine the nature of their antagonism or synergy [8,9]. Despite the significant progress made to date, currently available proteomic tools still lack the required sensitivity to detect peptides that are transient or in low abundance. Unfortunately, this applies to many signaling proteins, such as kinases and transcription factors, some of which have proven to act as key regulators during plant-pathogen interactions.

This review discusses the opportunities and challenges offered through the study of plant-pathogen interactions *in planta* using proteomic approaches to underline pathogen determinants of pathogenicity [4,10,11] and the resulting host defenses [12–14]. In particular, it highlights important findings in interactions involving fungi and oomycetes with biotrophic, necrotrophic, or hemibiotrophic lifestyles. Further to plant defenses, understanding pathogen counter-defenses could lead to better management of host resources towards durable resistance. Therefore, proteomic studies set to unravel the mechanisms that pathogens use to counter plant defenses are emphasized.

## 2. Proteomic Analysis of Plant Defenses and Pathogen Counter-Defenses

Proteomic approaches have been used to examine both pathogenicity determinants and the multilayered facets of plant defenses in many economically important diseases [15]. Studying both sides of the same equation is a legitimate attempt to assemble a more complete picture of the role of the detected proteome during the interaction. Such studies faced many challenges, *i.e.*, how to distinguish between plants and pathogen proteins and between those that are constitutive from those resulting from the interaction. However, further approaches had some success using tag labeling, subtractive methods, and differential treatments [4,10,16,17].

Analysis of the proteome of plant-pathogen interactions may serve many purposes through basic questions: (i) what plant proteins were activated in response to the pathogen and *vice-versa*? (ii) are these activations a result of new transcriptions/translations or post-translational activations of pre-formed inactive products? (iii) does the pattern of activation vary among pathosystems? (iv) is there any trend that could co-evolutionary explain defenses and counter-defenses and provide future prospects for engineering durable resistance? (v) could bioagents be used to accurately manipulate such interactions?

### 2.1. Proteomics as a Tool to Characterize Plant Defenses

Two types of plant defenses were defined; the pathogen-associated molecular patterns PAMP-triggered or innate immunity (PTI), and the pathogen effector-triggered immunity (ETI) [18]. PTI defenses are associated with the recognition of the pathogen followed by a series of downstream events. These include the alteration of ion fluxes across the host’s plasma membrane, the release of reactive oxygen species and nitric oxide as signaling molecules, the activation of MAP kinases, SA and JA/ET, and cross-talk between these pathways or with others such as ABA. ETI reactions, on the other hand, are elicited upon perception of effectors [19,20] released by the pathogen. Resistance gene-mediated defense follows, but often depends on the type of genes involved and the direct/indirect recognition of effectors [18]. Defense responses usually take place at sub-cellular levels, with cell structure rearrangements and transcriptional regulations that lead to the accumulation of defense-related molecules. The latter fortify the host cell wall [21–23], affect the integrity of the invading hyphae [24–26], and restrict progress of the pathogen [27,28]. Cell communication establishes among the tissues surrounding the initial infection sites and remote sites, hence leading to systemic acquired resistance (SAR) or induced systemic resistance (ISR) [29].

In host-pathogen interactions, proteomics have been truly complementary to functional genomics, and will continue to be so even more as entire genomes of plants and microorganisms are increasingly accessible. Vast Genbank databases with billions of sequences are daily being browsed, annotated or corrected/replaced by researchers from around the globe. It is interesting to note that proteomic investigation of plant defenses confirmed many of the results gathered using classical forward and reverse genetics, and biochemistry or molecular biology. It also provided a new generation of dataset, descriptive yet to be further analyzed and mastered for an overall view of the processes triggered in plants while fighting pathogens. It is possible through these approaches to now focus on an entire pathway involved in resistance and determine its interaction with other signaling and defense networks.

A number of pathosystems where proteomic approaches have been successfully used revealed either an increase or a decrease in protein abundance in response to infection by pathogens or to their pathogenicity factors. These proteins can be associated with photosynthesis and energy metabolism, anti-fungal activity, phytohormone homeostasis, ROS generation, detoxification and signaling, phenylpropanoid and lignin biosynthesis, and more (see below).

### 2.2. Proteomic Analysis of Plant Responses to Fungi and Oomycetes

Proteomic studies of plant-pathogen interactions *in planta* remain challenging. Data retrieval is important in determining proteins that accumulate differentially and are important for defense, pathogenicity, or counter-defense. Such studies often rely on (i) empirical comparison with databases and among pathosystems, (ii) complementary studies using the plant or pathogen individually as controls, or (iii) differential analysis using cultivars with various levels of resistance against highly aggressive isolates. Most studies, if not at a preliminary stage [4], clustered proteomes by nature, function, or sub-cellular localization of peptides [15,30], and reported on the technical challenges encountered in: (i) characterizing proteins in the absence of fully sequenced genomes of the host plants or their pathogens [31], and (ii) analyzing functions and regulation of the proteins encoded by the sequenced genomes.

To understand resistance of *Brassica carinata* to *Leptosphaeria maculans*, the causal agent of phoma leaf spot and stem canker, a proteomic investigation compared this species to a susceptible one, *Brassica napus* [16]. Forty-eight hours post-inoculation, 28 proteins accumulated differentially in the resistant phenotype, including superoxide dismutase, nitrate reductase, and carbonic anhydrase, as well as many photosynthesis-related enzymes. In cotton (*Gossypium hirsutum* L.), Coumans *et al.* [32] reported on proteins involved in plant defense against the black root rot fungus, *Thielaviopsis basicola*, especially PR-proteins and those implicated in ROS production and scavenging or in the isoprenoid biosynthesis pathway. In response to *Verticillium dahliae*, Wang *et al.* [33] also identified 51 upregulated and 17 downregulated proteins mainly involved in defense and stress responses, primary and secondary metabolism, lipid transport, and cytoskeleton organization. As such, analyzing proteomes during host-pathogen interactions will always generate differential expression of genes/proteins involved in disease or resistance. However, comparing proteomic research advances in pathosystems is complex, given the diversity of approaches used in proteome analysis and the genetic and biochemical backgrounds of the hosts or pathogens to be compared. Nonetheless, two categories can be distinguished, and involve either proteome data acquired only on the pathogen, or those gathered during host-pathogen interaction *in planta* [34]. One of the challenges relates to the distinction between proteins originating from the host and the pathogen. However, judicious experimental setups and recent technical and bioinformatics advances have been providing ways to better interpret such complex data. Data specifically related to the lifestyle of the pathogen, as biotroph, necrotroph or hemibiotroph are also valuable for proper result analysis and interpretation, but may bring their own complexities.

#### 2.2.1. Pathosystems Involving Biotrophs

Biotrophs establish in their host after they reach the plant surface, adhere and grow penetrating hyphae or appressoria, then haustoria [1]. Using these structures, CWDEs and effectors are released, some into the cells through mechanisms including injection, secretion, or simply diffusion and exocytosis [1,19,20,35]. Proteomic approaches developed in recent years revealed the content of these secretomes in some pathosystems. A growing number of proteins harboring RxLR or other conserved motifs have been determined to be invasive effectors essential for the injection of virulence factors into the host cells, hence leading to disease expression [19]. Effectors with LysM motifs are, on the other hand, thought to be defensive effectors [20,36] for the protection of the fungal cell-wall chitin layer, and preservation of its integrity. These two types of effectors are believed to be widespread among fungi, especially biotrophs.

Probably one of the most studied pathosytems involving a biotroph is the interaction between rice (*Oryza sativa* L.) and the blast pathogen *Magnaporthe grisea*. Past studies examined the proteome patterns in leaves interacting with the pathogen and under various levels of nitrogen [37,38]. Among the noticeable changes, 12 proteins including a PR-5 were up-regulated upon infection and differentially accumulated with nitrogen supplementation [12,13].

In a biotrophic interaction involving wheat lines and leaf rust pathogen *Puccinia triticina*, Rampitsch *et al.* [14] found no noticeable differences between soluble proteomes (pH 4–8) of inoculated and non-inoculated leaves of cultivar Thatcher, a susceptible line and Thatcher + Lr1, a near isogenic resistant one carrying *Lr1*, a dominant resistance gene to rust. To explain this, they suggested a masking effect of healthy tissues over the localized hypersensitive response in the resistant cultivar. In a similar approach, Devos *et al.* [39] reported many proteins related to defense, and to metabolic and cell differentiation processes that increased in response to inoculation of *Arabidopsis thaliana* with *Plasmodiophora brassicae*, the causal agent of clubroot in crucifers. The authors hypothesized that the establishment of typical meristematic zones around the infection site could be due to some of the proteins involved in cell differentiation, hence leading to the creation of a sink to host auxins, carbohydrates, nitrogen and energy to initiate gall formation and sustain pathogen growth and development. While studying changes in *Brassica napus* L. roots in response to compatible infections by *P. brassicae*, Cao *et al.* [40] revealed a decrease in the relative abundance of adenosine kinase. The latter has a role in cytokinin homeostasis as well as during infection of crucifers by *P. brassicae*. Lignin biosynthesis-associated caffeoyl-CoA *O*-methyltransferase and ROS-associated enzymes also decrease after infection, suggesting a reduction in cell wall strengthening, thus enhancing susceptibility. So far, more studies are needed to confirm the functions of many important proteins in these pathosystems, with a caution not to systemically extrapolate function findings onto other pathosystems. It is also paramount to cautiously design experiments that compare the proteomes of healthy and infected tissues, in order to avoid dilution of information such as that involving small hypersensitive spots in large leaf surfaces, and the use of appropriate checks.

Proteomic approaches were also used in plant-oomycete interactions. In the case of sudden oak death caused by *Phytophthora ramorum*, Meijer *et al.* [41] characterized 17 proteins that were exclusively secreted, including putative mucins, glucoside hydrolases, transglutaminases, an annexin-like protein and a Kazal-type protease inhibitor. These results confirmed findings in other oomycetes such as *Phytophthora cinnamomi* and *Phytophthora infestans*, known to produce spores with storage proteins [42] and a cocktail of CWDEs and suppressors [1,43–45]. These proteins are deposited in vesicles that are released during spore germination, thereby triggering the suppression of cell death and the hypersensitive response [46], the inhibition of superoxide generation [47], or accumulation of phytoalexins [43,45,48,49].

Changes in protein patterns of *Medicago truncatula*’s roots interacting with *Aphanomyces euteiches* [50] included cell wall proteins and enzymes involved in the phenylpropanoid pathway, especially those associated with isoflavonoids and phytoalexin biosynthesis [51]. Similarly, Curto *et al.* [52] and Amey *et al.* [53] identified leaf proteins implicated in pea resistance to powdery and downy mildews, caused by *Erysiphe pisi* and *Peronospora viciae*, respectively. Therefore, many similarities exist between systems involving biotrophic fungi and oomycetes, which is not surprising due to their similar lifestyle.

#### 2.2.2. Pathosystems Involving Necrotrophs

As with biotrophs, the data available so far for necrotrophs is scattered among different levels of tissues and experimental setups. Leaf proteome of rice after inoculation with *Rhizoctonia solani* showed an increase in the relative abundance of proteins involved in energy metabolism and photosynthesis, defense, antioxidant activity, as well as protein unfolding and degradation processes [54]. In maize embryos inoculated with *Fusarium verticillioides*, proteomic host defense responses included PRs, ROS-detoxifying enzymes, and others implicated in protein biosynthesis or folding and stabilization processes [55]. In wheat infected by *Fusarium graminearum*, which causes Fusarium head blight, Zhou *et al.* [17] ranged 33 plant proteins into defense-related or metabolism-related groups, and more have been identified more recently in wheat and barley interactions with this pathogen [56,57]. In response to *Alternaria brassicae*, *Brassica napus* L. produced enzymes involved in ROS production as well as auxin signal transduction and metabolic pathways [58], while application of oxalic acid, a major pathogenicity factor of many necrotrophic pathogens, including *Sclerotinia sclerotiorum*, resulted in down-regulation of SA-dependent pathways and up-regulation of JA/ET- or ABA-dependent ones [59,60].

On the pathogen side, the grey mold fungus *Botrytis cinerea* has been greatly investigated in recent years using an array of approaches, including proteomics [61], to identify its pathogenicity factors [62]. It was recently shown to manipulate the network of phytohormones and oxidative burst system of the host [1,63]. Comparison of proteomic profiles of two isolates of this fungus, with contrasting levels of virulence [61], showed their ability to differentially accumulate proteins, including isoforms of malate and glyceraldehyde-3-phosphate dehydrogenases.

#### 2.2.3. Pathosystems Involving Hemibiotrophs

*Fusarium oxysporum* f.sp. *lycopersici* induced more defense-related proteins in the xylem sap of tomato plants in the compatible interaction than in the incompatible one and the non-infected checks [64]. Using the same model, Houterman *et al.* [31] identified 33 proteins, among which 21 were from tomato and seven from the pathogen, with 13 ascribed to response to infection, and others identified as CWDEs, peroxidases, and proteases.

Many of the proteins identified in such interactions may have homology in different plant species, but definitive confirmation always requires further functional analyses. Some level of difficulty may arise with regard to the fungal *versus* plant origin of identified proteins, and in functional analyses in systems where transformation is not straightforward, such as wheat.

### 2.3. Proteomics as a Tool to Examine Pathogenicity Determinants

Plant pathogens specialize in attacking their respective hosts and feeding on their tissues, thus causing disease and, potentially, epidemics. They possess a large number of features that allow them to infect and establish in/on plant tissues [1,65,66]. Upon infection, necrotrophs release an array of enzymes that allow them to dispose of the organic matter of dead tissues [67,68]. Biotrophs, on the other hand, require their host cells to remain alive and have evolved other pathogenicity determinants, such as haustoria, following host cell penetration [1]. Similarly, pathogens producing toxins may secrete these molecules at the forefront of the infection site to lay the ground for mycelial invasion [65,66,69–71]. Recently, advances in understanding the mechanisms of biotrophic, necrotrophic and hemibiotrophic lifestyles were reviewed [72], with special emphasis on the biotrophy-necrotrophy switch of hemibiotrophic pathogens. During the last few years, a new field of research focusing on pathogen effectors has emerged [19,20]. Pathogen effectors enable plant defense suppression and can reprogram the metabolic machinery of infected tissue to benefit the pathogen in its growth and development [73].

Effectors were deemed to be widespread among invading pathogens and can be either invasive or defensive [74,75]. Invasive effectors allow a given pathogen to attack, *i.e.*, polygalacturonases [4,68], whereas, the defensive ones protect it from degrading enzymes (*i.e.*, chitinases; [20]) and toxic metabolites synthesized by the host (*i.e.*, phytoalexins and defense-related metabolites; [2,21,22]). Suppressors of plant defenses are also among the pathogenicity determinants/effectors in fungi and oomycetes as they were documented in bacteria and viruses [1]. Biotrophs needing to establish a trophic relationship with their live host cells have evolved these type of mechanisms to temporarily turn off the plant guards and defenses and avoid detection [1]. For hemibiotrophs and nectrotrophs, more sophisticated strategies to suppress defense mechanisms have recently been reported. These include the manipulation of signaling pathways [2,63], the hijacking of essential defense molecules [76], the detoxification of major defense-related metabolites [21,22] and others yet to be determined. In a recent study involving *V. dahliae*, a fungus with multiple lifestyles, varying from necrotroph, to hemibiotroph, to endophyte, on a wide host range [77], El-Bebany *et al.* [4] established a proteomic map (pH 4–7) using *in vitro-*cultures of weakly- *versus* highly-aggressive isolates. The comparison revealed 25 differentially-regulated proteins, most of which were important determinants for host penetration and colonization, development of microsclerotia, and resistance to stress and antibiotics in the highly-aggressive isolate tested. The authors also reported on an increase in protein abundance of the isochorismatase hydrolase, thought to be implicated as a suppressor of plant defense responses and signaling [4]. Proteome maps are also being developed for other important fungi, such as *Cladosporium fulvum* and *Mycosphaerella fijiensis*, the causal agents of leaf mould in tomato and black Sigatoka in bananas and plantains, respectively. Four isolates of *Verticillium albo-atrum* from two geographical zones were used to compare the proteomes of two pathotypes on hop and establish the first mycelial proteome map of this fungus [10]. Among the 53 proteins identified, 17 were ascribed functions, and about 30 differentially accumulated in the mild *versus* the lethal pathotype. Among the proteins differentially abundant in the lethal pathotype were peroxiredoxin and ascorbate peroxidase, cytoskeleton components and regulators, and proteins involved in protein synthesis and energy metabolism.

In *L. maculans*, which causes economically important diseases of crucifers, including canola blackleg and stem canker [23,78], the mycelial proteome has recently been reported [79], showing several CWDEs previously identified in this and other necrotrophs. Proteomic analysis of mycelia provides important insights into the ability of pathogens to evolve new tactics to deal with plant defenses. However, there is still much to achieve regarding the spatio-temporal accumulation of peptides/proteins produced by pathogens throughout their disease cycle, and how such choices allow them to strategically take advantage of specific plant weaknesses, or surmount most sophisticated plant defenses. It is nonetheless obvious that integration of proteomic, transcriptomic, and metabolomic data over time is a major step towards understanding such intricate interactions. In their study on exoproteome of the notorious nectrotrophic fungus *Sclerotinia sclerotiorum*, Yajima and Kav [67] reported on 52 secreted proteins that included many CWDEs, known as pathogenicity factors in this pathogen, and highlighted the complementarity of proteomics to genomic approaches by identifying an α-l-arabinofuranosidase that was omitted in functional genomic studies involving ESTs.

Cao *et al.* [11] examined the proteome profile of virulent and avirulent isolates of the biotrophic *Pyrenophora tritici-repentis*, the causal agent of tan spot in wheat, known for its ability to produce several Ptr host-specific toxins. Virulent isolates usually produce the toxic chlorosis-inducing protein Ptr ToxB due to the expression of the *ToxB* gene. Avirulent isolates harbor a homolog of the gene but do not produce a functional protein [69,80]. Aside from *ToxB* gene, the virulent isolate actually exhibited a distinctive profile where several translated and accumulated proteins were up-regulated and ascribed to virulence, including an α-mannosidase and an exo-β-1,3-glucanase. In addition, saprophytic growth and development may account for differences in the proteome profile between virulent and avirulent isolates.

In other filamentous fungi, a proteome map has been created from mycelia collected during the dimorphic transition from budding to filamentous growth in *Ustilago maydis* [81]. In *Blumeria graminis* f.sp. *hordei*, proteic fractions collected from conidiospores were used to generate such a map. Among the identified proteins were enzymes involved in carbohydrate, lipid, and protein metabolism [82]. In *Cladosporium fulvum* interacting with tomato, three extracellularly secreted proteins, namely CfPhiA, Ecp6 and Ecp7, have been recently characterized [83]. Therefore, proteome characterization in this class remains sporadic and many studies are needed to fill the gaps for the different development stages of oomycetes and during their interactions with their hosts.

In oomycetes, especially *Phytophthora* spp., proteomics are informative in studying the proteic pool during various stages of growth and development, including hyphae, cysts, sporangia, zoospores, and appressoria [84–86].

Some work has been done on the use of exogenous supplementation of chemically-synthesized molecules. Shimizu *et al.* [87] used vanillin on *Phanerochaete chrysosporium* cultures and reported on the identification of up-regulated proteins involved in vanillin metabolism. A similar procedure was applied on the white rot fungus, *Phanerochaete chrysosporium*, using benzoic acid [88], allowing the characterization of a pool of mycelia proteins differentially responsive to the treatment. In general, not many fungal/oomycete pathogens have been extensively studied in this respect. A recent review reports on *B. cinerea*, *S. sclerotiorum*, and *F. graminearum* as the most studied plant pathogenic fungi in proteomics [89].

The studies available so far highlight the worth of proteomic approaches in determining pathogenicity factors and effectors of many pathogens, but also suggest that a great horizon is open to more studies to investigate the function and roles of individual microbial peptides and proteins in development stages and host-pathogen interactions.

## 3. The Future of Proteomics in Studying Plant-Pathogen Interactions

Major discoveries in plant-pathogen interactions using proteomics are yet to come, with the development of high throughput and sophisticated procedures to analyze, annotate, and compare proteomes [90], including translational plant proteomics [91]. Advances are to be expected with the development of new tools such as liquid-phase IEF, high resolution 2D, MRMs and iTRAQ quantification procedures [92,93]. Progress is also being made in mathematical algorithms and statistical procedures to cluster and analyze massive datasets. Accurate linking of functions to targets is being sought, and will still be, while studies are moving from pathosystems involving model species, such as the thale cress, to economically important crops and their diseases. Metaproteomics are also being developed to examine cohabitant microbial species proteomes, their interplays, and understand relationships among microorganism communities present either in the rhizosphere or the phyllosphere. This will lay the ground for the application of proteomic approaches in studying tripartite relationships, involving the host, its pathogen, and biocontrol agents or other biological entities. The accruing information about pathogens that evolve strategies to counteract plant defenses will certainly benefit from proteome studies and help understand intricacies of the co-evolution between pathogens and their hosts.

### 3.1. Going beyond the Model Systems

Proteomic approaches have not yet provided a full coverage of the entire proteome of plant species such as *A. thaliana* or the model fungus *C. fulvum*. More than 35,000 translated proteins were initially predicted in *A. thaliana* and currently only around 1/10 of that number has been characterized [94], likely due to challenges in separating and identifying all the proteins expressed under specific conditions. The latter relate to insolubility, low abundance, size, or pH. However, proteomics findings often feed back to genomics by complementing the annotation of ORFs and correcting gene structure predictions. In addition, proteomics in model plant species allows for the determination of recurrent peptides to be used as internal controls for large-scale proteomic studies. It also allows for the examination of post-translational processes that proteins go through, such as cleavage of signal peptides to allow compartmentalization, *N-*glycosylation, or phosphorylation. Such knowledge accelerates our understanding of other pathosystems involving economically important crops and destructive pathogens, hence increasing the likelihood of setup control management strategies that reduce their impact and restore yield and quality.

Carpentier *et al.* [95] discussed the challenges and opportunities facing proteomic approaches in non-model species, such as bananas and plantains, where the proteome analysis has flourished in absence of sequencing data on a large portion of the genome and with enormous challenges in conducting large scale gene expression. El Hadrami *et al.* [66] had examined the isoenzymatic profile and activities of a set of cytosolic and chloroplastic proteins involved in the scavenging and detoxification of ROS in a susceptible *versus* a partially resistant banana cultivar interacting with the toxin juglone, one of the pathogenicity factors of *M. fijiensis*. The main difference between the two tested phenotypes was in the quickness in triggering their antioxidant systems on perceiving the toxin, and how long the system remains active and coordinated.

It is clear that in the years to come, a continuous move will occur from studying plant models to studying non-model species. In spite of the lack of extensive genomic data on the latter and/or how they differ from model species, they will still benefit from the available genomics and proteomics datasets. There is a need for appropriate extraction methods that minimize interferences with other analytes and maximize separation and quantification. As for identification and assigning functions, one would still rely on the background of model species unless other information can be gathered through complementary approaches.

### 3.2. Resolving Unambiguous Protein Identification

Significant progress has been made in proteomic studies in many pathosystems using differential models or comparing inoculated with mock-inoculated controls [4,10,14,33]. Separation of the proteome is conducted by 1D or 2D-PAGE *i.e.*, [4,10], which often lack sensitivity and reproducibility [10]. The advent of nanoflow-LC technology has made it possible to minimize some of these issues *i.e.*, [4]. To enhance the separation and gain an in-depth understanding, new approaches, such as liquid-phase IEF coupled to high-resolution 2D, have also been used in fungi [79]. Immense challenges are still encountered in mass spectrometry detection, identification, and quantification. Current methods use nanoflow-LC coupled to mass spectrometry technologies with triple or quadruple quads such as MALDI, MALDI-Q-TOF, ESI-MS/MS, ICP-MS/MS, as well as programs specialized in data acquisition, spectral analysis and protein identification through matches with onsite or online databases (*i.e.*, MASCOT, SEQUEST, X!TANDEM). Other quantification procedures using stable labeling of ^13^C, ^2^H, ^15^N, or ^18^O within techniques such as ICAT [96–99], SILAC [100], and iTRAQ™ [101] have also became popular and have greatly improved the quantitative determination of the detected proteins. iTRAQ MS/MS technology has been applied in profiling differentially translated and accumulated proteins from *F. graminearum* after an *in vitro*-induction of mycotoxins synthesis in comparison with a mock-treated control [102]. The presence of phosphorus and sulfur residues in proteins has also been used along with technologies such as ICP-MS to translate the molarity of these residues into absolute protein concentrations.

Resolving unambiguous identification of proteins among contrasting treatments remains an important challenge in proteomics. While one can conceivably manually compare various gels with a limited number of differentially up- or down-regulated protein spots, difficulties arise when proteome analysis is conducted using high-resolution 2D-PAGE, especially after the use of liquid-phase IEF or other pre-screening methods. Theoretically, on these gels, up to 15,000 protein spots could be detected but practically only a third of that number or less can accurately be resolved and analyzed. An extensive development of hardware and software to precisely analyze these images has been seen in recent years to maximize spot annotations, establish protein patterns, resolve coalescent spots, and provide an accurate comparison among tested treatments. To further minimize identification errors, spots can be excised from the PAGE and enzymatically digested and subjected to MS/MS and database matching. Matching against available databases is often hampered by an extensive presence of hypothetical proteins with no assigned function and/or with lack of full protein sequences. Combining transcriptomic and proteomic approaches often yield an accurate correlation between the variation in the gene expression and the changes in the levels of the detected proteins, allowing for an exact determination of identity and function [2]. These can also be complemented with an array of *in silico* analysis to predict compartment localization, structures, and function [2]. In addition, a better knowledge of the pathosystems considered for proteomic analysis through other molecular, biochemical, physiological, and epidemiological approaches allows and eases the unambiguous interpretation of the gathered data.

### 3.3. Assigning Functions to Detected Proteins

#### 3.3.1. Searching PAMPS

Proteome analysis is currently becoming an important component in dissecting plant-pathogen interactions, and represents a substantial addition to the daily-basis research tool box. It helps identifying up- or down-regulated proteins and assigned functions that are linked to pathogenicity, defense, or counter-defense. The gap in access to pathogen genomes will eventually be filled with the arrival of faster new-generation sequencers. This will balance the former research trend that mainly focused on the elucidation of plant defenses. A number of effectors, Avr genes and proteins as well as PAMPs have already been characterized [18,20]. A new flow of information gathered from the characterization of these proteins is currently being used to assess their roles in pathogenicity, in inducing susceptibility or HR in the host, or even in manipulating host signaling cascades and overall defense-responses. Proteomic studies have recently characterized a new class of PAMPs, from the oomycete *P. parasitica* var. *nicotianae*, with a cell wall anchored position and an ability to bind cellulose [103]. Similarly, the approach revealed other PAMPs from oomycetes and other fungi [104,105].

#### 3.3.2. Characterizing Membrane Proteins and Receptors

The development of proteomics had taken protein-protein interactions to a level above the usual yeast two-hybrid systems. It is now possible to isolate membrane proteins and general or specific receptors [90]. Membrane proteins and binding receptors achieve their physiological functions through interaction with other intracellular proteins and effectors. More exploration could lead to the characterization of multiple proteins and complexes interacting either directly or indirectly with intercellular effectors and proteins to trigger signaling cascades during plant-pathogen interactions. Santoni [106] described a two-step procedure to recover transmembrane proteins of the plasma membrane of *A. thaliana*. First, the author depleted the plasma membrane from its soluble cytosolic contaminants and other functionally associated proteins, using an alkaline treatment, then, solubilized the hydrophobic remaining proteins. Similarly, Sazuka *et al.* [107] used a proteomic approach on the same plant to identify membrane-bound proteins. Analysis of such proteins revealed 64 informative spots belonging to 42 protein families including transporters, channels, receptors, and membrane-anchored proteins such as GTPases. Elortza *et al.* [108] analyzed the membrane-embedded proteins involved in glycosylphosphatidylinositol metabolism and signaling pathways. These proteins are believed to play a role in cell adhesion and perception of effectors and in signal transduction during host-pathogen interactions. Biological membranes are predicted to contain 30% of proteins so far, but the difficulties arising from their poor solubility, separation and identification will greatly benefit from advances in proteomics [109], which may lead to a better understanding of their functions and roles in plant-pathogen interactions.

#### 3.3.3. Unveiling Wound-Healing Processes

In comparison with the biomedical sector, there seem to be fewer opportunities from current knowledge to minimize plant infection and colonization through wound-healing based on proteome analysis. Studying membrane proteins using proteomic approaches is rather difficult, although new advances in analytical methods in the next few years may help overcome such challenges. A strategy to accelerate wound-healing would lead to limiting plant infection by some pathogens. Slicing a potato tuber triggers natural wound-healing, which makes this species a useful model that was explored using proteomics [110,111]. Proteomics revealed a substantial alteration of the proteome pattern [110], including suberization-associated anionic peroxidases, thought to play a major role reconstructing the damaged periderm. While analyzing the tuber epidermis proteome in wounded *versus* non-wounded tubers, Barel and Ginzberg [111] showed that wounding enhances the translation and accumulation of proteins with a role in tissue differentiation and proliferation and in the oxidative respiratory chain and energy metabolism. Among the proteins detected were caffeoyl-CoA *O*-methyltransferase and a series of peroxidases, known for their role in potato periderm suberization [112]. This knowledge could well be used for creating cultivars with accelerated wound-healing process that could minimize their exposure to attacks by necrotrophic or soil-borne pathogens.

#### 3.3.4. Examining Further the Roles of Fungal and Oomycete Effectors

One of the fields that will tremendously benefit from the expansion of proteomics is the identification of effector proteins [20] and their receptors. Extracellular proteins (Ecps) have been purified and characterized from apoplastic fluids of tomato plants subjected to infection with *C. fulvum*. These proteins are widespread among tested pathogen isolates, and exhibit similar properties as race-specific Avr elicitors with regard to host perception [20]. Similarly, proteomic approaches have been developed to investigate the RxLR effectors and other similar motifs that are thought to be omnipresent across fungi and oomycetes. Using proteomics, it has been possible to show that a positive selection has targeted the *C*-terminal of these effectors, thus contributing to their evolution and diversity [113]. This is in agreement with the current knowledge about these effectors, where the *N*-terminal domain is known to be involved in their secretion, whereas the *C*-terminal domain is implicated in interference with plant defenses once they are inside the host cells. Another category of effectors that has been getting increasing attention in recent years is the so-called LysM, believed to be implicated in the protection of the chitin layer of the fungus being targeted by plant chitinases and in modulating plant defenses [20]. In the next few years, proteomics should provide a better understanding of their mode of action, their roles in pathogenicity, and their implications to interfere with plant defenses.

#### 3.3.5. Deciphering Regulons and Operons

The notions of transcriptional operons and proteome regulons are currently gaining ground after the discovery, through genomic and proteomic approaches, of a vast interplay and connectivity between the genes, proteins and signaling pathways that operate in plant-pathogen interactions. Operons represent clusters of genes that are functionally interrelated and co-regulated by the same promoter [114]. Regulons are clusters of genes that are under the same regulatory mechanisms of a single or multiple proteins [115,116]. Gene duplication that occurred during evolution had led to the setting of these gene clusters into paralogous and orthologous dispositions either among the core- or the plastic-genome [117]. The current advances in proteomics, in parallel with genomics, and metabolite profiling, are in the process of providing a better understanding of the mechanisms of such complex networks, especially during plant-pathogen interactions [118].

### 3.4. Studying Tripartite Interactions

Pathosystems involving a host, a pathogen, and a biocontrol agent are more challenging to study in terms of discerning the role of each one of them in the interaction. Proteomics-based approaches have been suggested to study these complex systems [119] and unveil the three-way signaling cross-talks. Marra *et al.* [119] used an experimental set-up that allowed them to separate the individual proteomes from beans, *B. cinerea* or *Trichoderma solani* and the antagonistic biocontrol agent *Trichoderma atrviridae*. The approach was instrumental in showing an up-regulation of bean’s defense-related proteins such as PRs and that *T. atroviride* qualitatively and quantitatively alters the protein profile of the plant regardless of infection by a pathogen. The study also revealed proteins in *T. atroviride* with homology to fungal hydrophobins and ABC transporters, and the up-regulation in the pathogen proteome of virulence factors such as cyclophilins, when interacting with the host alone or in the concomitant presence of the biocontrol antagonist as well. Many other studies need to be investigated in such an integrated mode in order to gather more valuable information that is closer to the events that happen in nature.

### 3.5. Characterizing Pathogen Counter-Defenses

Between pathogens and their hosts, the balance was for many decades leaning towards studying plant defenses. In recent years, with the advent of genomics-based technologies that made more sequenced pathogen genomes available, more studies burgeoned on characterization of pathogenicity factors and pathogen-based strategies that avoid host resistance. Such strategies included ways to avoid detection by the host, suppress or cope with host defenses, and strategically manipulate host pathways and signaling cascades, thereby leading to unnecessary and energy-costly defenses or ones more beneficial to the pathogen [1,2]. These seem to be evolutionary strategies for co-existence that allow the pathogens to make the best of their trophic interactions with the hosts. While many biotrophs seem to have adapted strategies to either remain undetectable by plant cognate guardees and PRRs or to cope with plant defenses by detoxifying phytoalexins and other defense-related metabolites [1,21,22], hemibiotrophs and necrotrophs tend to raise the bar in sophisticating their counter-defense strategies [1,2,63]. For instance, necrotrophic fungi such as *S. sclerotiorum* suppress the host’s ROS signaling cascade *in planta* via catalases, superoxide dismutases, or other scavenging molecules, such as mannitol or oxalic acid [120,121], hence leading to an enhanced hypersensitive response locally and/or preventing signal transduction to remote healthy sites. *Botrytis cinerea* forces its host to produce ROS, which kill the tissues, thereby enabling their colonization [122,123]. *Fusarium oxysporum*, on the other hand, has been reported to use the COI1-mediated JA-signaling to promote disease development in *A. thaliana* [76]. This manipulation of signaling pathways has also been shown in potato interacting with *V. dahliae* and tomato with *B. cinerea* [2,63]. In potato, plant defenses include an increase in the synthesis and accumulation of rutin. This defense-related flavonol-glycoside represents a carbon source for the pathogen, but cleaving the sugar moiety, using enzymes such as glucosidases and rhamnosidases, exposes *V. dahliae* to quercetin, the rutin’s aglycone. During its coevolution with its hosts, *V. dahliae* has evolved a mechanism that allows it to detoxify this flavonol using its quercetinases [2]. The byproduct of detoxification, the 2-PCPGCA, is composed of phloroglucinol, a potent antibiotic that theoretically expands the pathogen’s competitiveness in the rhizosphere, and protocatechuates that get converted into benzoates and salicylates, potential triggers of the SA-pathway, known to interfere with the JA-pathway. In the years to come, these reports on counter-defense strategies evolved by hemibiotrophs and necrotrophs will benefit to unveil new mechanisms and interplays using proteomics-based technologies.

## 4. Concluding Remarks

In recent years, proteomic analysis has enhanced our knowledge about plants, pathogens and their interactions with each other and with other components of their respective environments. It also complemented information gathered through forward and reverse genetics, and functional genomics. The increasing availability of fully-sequenced and annotated genomes allowed the making of great strides towards identification and establishment of protein functions, which consequently feeds back on accurate annotation of ORFs and prediction of gene functions. Proteomics have also made it possible to study protein-protein interactions, characterize membrane-anchored proteins, and discover new PAMPs. In plant-pathogen interactions, proteomic approaches are instrumental in deciphering plant defense pathways and signaling cascades, and unveiling pathogenicity factors and pathogen effectors. The field is set to generate even more opportunities, but not without more complexity, in studies meant to better understand (i) tripartite interactions where the host plant interacts with both beneficial and pathogenic microbes, and (ii) the strategies evolved by pathogens to counteract their host defenses and other microbes’ attacks. Hopefully, integration of this knowledge from complex interactions will set us a step ahead towards developing more novel and environmentally-safe disease management strategies.

## Abbreviations

1 or 2D-PAGE: 1- or 2-dimensional polyacrylamide gel electrophoresis
2-PCPGCA: 2-protocatechoylphloroglucinolcarboxylic acid
ABA: Abscisic acid
CWDEs: cell-wall degrading enzymes
ESI-: Electrospray ionization
ET: Ethylene
ICAT: isotope-coded affinity-tag
ICP-: Inductively coupled plasma
IEF: Isoelectrofocusing of a protein
ISR: systemic acquired resistance
iTRAQ™: isobaric tags for relative and absolute quantification
JA: Jasmonic acid
LC: liquid chromatography
MALDI: Matrix-assisted laser desorption/ionization
MRM: Multiple Reaction Monitoring
MS/MS: tandem mass spectrometry
*m/*z: mass over charge
ORFs: open reading frames
PGPRs: Plant Growth-Promoting Rhizobacteria
PAMPs: pathogen-associated molecular patterns
PRs: pathogenesis-related proteins
Q-TOF: Quadrupole time-of-flight
ROS: Reactive Oxygen Species
SA: salicylic acid
SAR: systemic acquired resistance
SILAC: stable isotope labeling by amino acids in cell cultures
